# Lessons from the reestablishment of Public Health Laboratory activities in Puerto Rico after Hurricane Maria

**DOI:** 10.1038/s41467-019-10776-y

**Published:** 2019-06-20

**Authors:** Margaret C. Hardy, Rita C. Stinnett, Kristine J. Kines, Danisha M. Rivera-Nazario, David E. Lowe, Alexandra M. Mercante, Nathalie Gonzalez Jimenez, Rosa I. Cuevas Ruiz, Héctor I. Rivera Arbolay, Rafael L. Gonzalez Peña, Mayra Toro, Alma A. Trujillo, Claudia L. Pappas, Anna C. Llewellyn, Francisco Candal, María Burgos Garay, Gerardo A. Gomez, Jeniffer Concepcion Acevedo, Marisela Ansbro, Hercules Moura, Michael W. Shaw, Atis Muehlenbachs, Lovisa C. Romanoff, Brittany J. Sunshine, Dale A. Rose, Anita Patel, Craig N. Shapiro, S. Carolina Luna-Pinto, Satish K. Pillai, Eduardo O’Neill

**Affiliations:** 1 Laboratory Leadership Service Fellowship, Centers for Disease Control and Prevention, Atlanta, GA 30329 USA; 2 Division of Foodborne and Waterborne Diseases, National Center for Emerging and Zoonotic Infectious Diseases, Centers for Disease Control and Prevention, Atlanta, GA 30329 USA; 3 Division of Viral Diseases, National Center for Immunization and Respiratory Diseases, Centers for Disease Control and Prevention, Atlanta, GA 30329 USA; 4 Division of Parasitic Diseases and Malaria, Center for Global Health, Centers for Disease Control and Prevention, Atlanta, GA 30329 USA; 5 Division of Laboratory Sciences, National Center for Environmental Health, Centers for Disease Control and Prevention, Atlanta, GA 30329 USA; 6 Division of High-Consequence Pathogens and Pathology, National Center for Emerging and Zoonotic Infectious Diseases, Centers for Disease Control and Prevention, Atlanta, GA 30329 USA; 7 Public Health Laboratories, Puerto Rico Department of Health, San Juan, PR 00936 USA; 8 Division of Vector-Borne Diseases, National Center for Emerging and Zoonotic Infectious Diseases, Centers for Disease Control and Prevention, Atlanta, GA 30329 USA; 9 Influenza Division, National Center for Immunizations and Respiratory Diseases, Centers for Disease Control and Prevention, Atlanta, GA 30329 USA; 10 Global Immunization Division, Center for Global Health, Centers for Disease Control and Prevention, Atlanta, GA 30329 USA; 11 Office of Technology and Innovation, Office of Science, Centers for Disease Control and Prevention, Atlanta, GA 30329 USA; 12 Division of Healthcare Quality Promotion, National Center for Emerging and Zoonotic Infectious Diseases, Centers for Disease Control and Prevention, Atlanta, GA 30329 USA; 13 Division of Select Agents and Toxins, Center for Preparedness and Response, Centers for Disease Control and Prevention, Atlanta, GA 30329 USA; 14 Office of the Deputy Director for Infectious Diseases, Centers for Disease Control and Prevention, Atlanta, GA 30329 USA; 15 Division of Preparedness and Emerging Infections, National Center for Emerging and Zoonotic Infectious Diseases, Centers for Disease Control and Prevention, Atlanta, GA 30329 USA; 16 Division of Emergency Operations, Center for Preparedness and Response, Centers for Disease Control and Prevention, Atlanta, Georgia 30329 USA; 17 Influenza Coordination Unit, National Center for Immunizations and Respiratory Diseases, Centers for Disease Control and Prevention, Atlanta, GA 30329 USA; 18 Office of the Director, Center for State, Tribal, Local and Territorial Support, Centers for Disease Control and Prevention, Atlanta, GA 30329 USA; 19 Present Address: Office of the Chief Regulatory Scientist, Australian Pesticides and Veterinary Medicines Authority, Armidale, New South Wales 2350 Australia; 20 Present Address: Office of the Deputy Director for Infectious Diseases, Centers for Disease Control and Prevention, Atlanta, GA 30329 USA; 21 Present Address: Division of Preparedness and Emerging Infections, National Center for Emerging and Zoonotic Infectious Diseases, Centers for Disease Control and Prevention, Atlanta, GA 30329 USA

**Keywords:** Natural hazards, Public health

## Abstract

Public Health Laboratories (PHLs) in Puerto Rico did not escape the devastation caused by Hurricane Maria. We implemented a quality management system (QMS) approach to systematically reestablish laboratory testing, after evaluating structural and functional damage. PHLs were inoperable immediately after the storm. Our QMS-based approach began in October 2017, ended in May 2018, and resulted in the reestablishment of 92% of baseline laboratory testing capacity. Here, we share lessons learned from the historic recovery of the largest United States’ jurisdiction to lose its PHL capacity, and provide broadly applicable tools for other jurisdictions to enhance preparedness for public health emergencies.

## Abstract (web summary)

Hurricane Maria hit Puerto Rico in 2017 and resulted in a complete loss of activity of the Public Health Laboratories. Here, the authors discuss the approach taken and tools developed to re-establish activity in these laboratories using a quality management system and the lessons learned in this process.

## Introduction

**Fig. 1:**
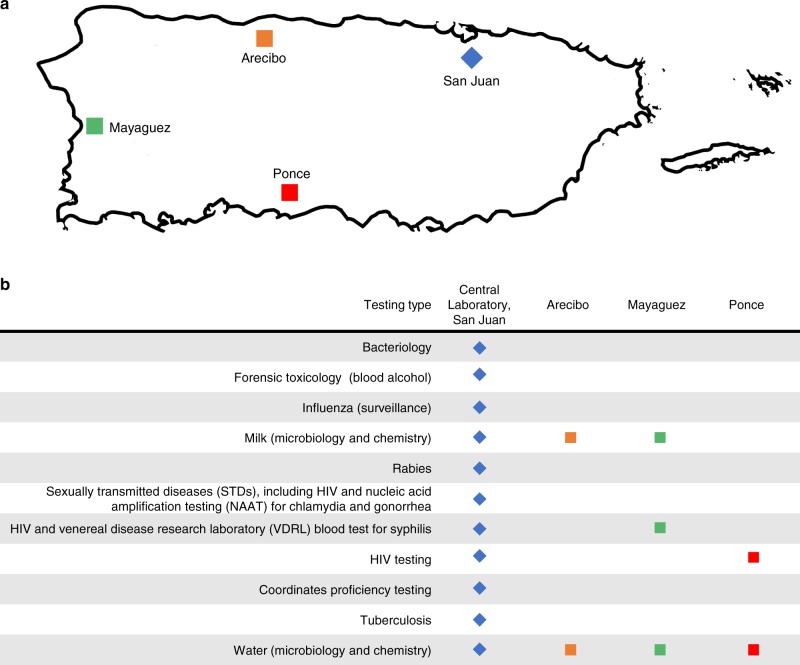
Description of the Public Health Laboratories in Puerto Rico. The locations (**a**) and the types of testing provided (**b**) are presented. This figure is derived from a map originally released into the public domain and available in the Wikimedia Commons repository (https://commons.wikimedia.org/wiki/File:Map_of_Puerto_Rico_highlighting_Desecheo_Island.svg)

In September 2017, Hurricane Maria severely damaged the Puerto Rico Department of Health (PRDH) Public Health Laboratories (PHLs), resulting in immediate and long-term impacts on public health services. PHLs serve an integral role in protecting human health by conducting laboratory tests to identify disease outbreaks and monitor the safety of food, water, and the environment. In addition, the Puerto Rico PHLs partner with the Laboratory Response Network (LRN; https://emergency.cdc.gov/lrn), a US network of military, local, state, and federal laboratories (including a number of international laboratories) that can respond to many public health emergencies. PRDH served a population of more than 3.3 million people before Hurricane Maria, and the storm represented a historic loss of capacity for the most populous jurisdiction in the United States to completely have lost its PHL operations. The PRDH PHL system consists of four facilities across the island, located in the municipalities of San Juan, Arecibo, Mayaguez, and Ponce (Fig. 1). Each location houses between one and eight individual laboratories responsible for pathogen surveillance and diagnosis (San Juan, Ponce, Mayaguez), environmental water testing (all), and milk testing (San Juan, Mayaguez, Arecibo); the central laboratory in San Juan provides support to the three regional facilities (in Arecibo, Mayaguez, and Ponce) and administers the Puerto Rico Proficiency Testing Program (PTNET), which supports more than 930 clinical laboratories responsible for diagnostic and surveillance testing across the island (http://ptnet.salud.gov.pr). Of note, a number of individual laboratories in the central, Mayaguez, and Ponce facilities are under strict regulatory oversight by the Centers for Medicare & Medicaid Services (CMS) as this agency is responsible for the Clinical Laboratory Improvement Amendments (CLIA). However, this agency issued a waiver to regulatory requirements. The PRDH PHL system also includes the Biological and Chemical Emergencies Laboratory (BCEL) and the Newborn Screening Laboratory, both of which are located in San Juan.

As part of the CDC Emergency Operations Center (EOC), the CDC Laboratory Team (consisting of 23 Spanish-speaking responders from October 2017 through May 2018) worked with laboratory subject matter experts (SMEs) at PRDH and began efforts to reestablish laboratory activities to pre-Hurricane Maria levels in coordination with federal, state, and non-governmental partners. The main goal of this work was to facilitate the reestablishment of full laboratory testing capacity at PRDH. This Perspective describes the significant challenges faced in restoring laboratory functions, capacity, services, and clinical testing in Puerto Rico, the specific efforts that were needed after the event and the lessons learned in this process.

**Fig. 2:**
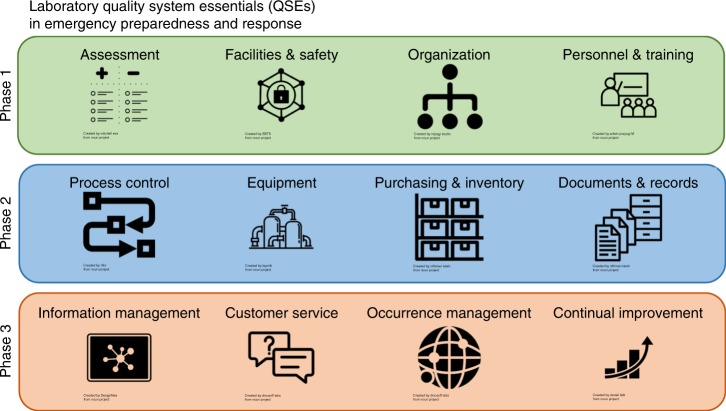
The 12 laboratory quality system essentials (QSEs). QSEs were used as a useful framework for an emergency response. Phase 1 included assessments, facilities and safety, organization and personnel training; Phase 2 included process control, equipment, purchasing an inventory and documents and records; phase 3 included Information management, customer service, occurrence management and continual improvement. The component images in this figure are from The Noun Project under a CC-BY 3.0 license, “Assessment” icon by Mitchell Eva, “Facilities and Safety” by SBTS, “Organization” by inipagi studio, “Personnel & Training” by Edwin Prayogi M, “Process Control” by Rflor, “Equipment” by Laymilk, “Purchasing & Inventory” and “Documents & Records” by Nithinan Tatah, “Information Management” by DesignNex, “Customer Service” and “Occurrence Management” by Dinosoft Labs, and “Continual Improvement” by Daniel Falk

The team’s approach aligned with principles outlined by the Clinical and Laboratory Standards Institute (CLSI) as laboratory quality system essentials (QSE) (Fig. 2)^1^. Briefly, Fig. 2 details the three-phase approach which began in Phase 1 with laboratory assessments to understand the extent of the damage to facilities, the risk to personnel and organize recovery efforts. The actual implementation of recovery activities fall in Phase 2 with recalibration, validation or replacement of equipment, supplies and reagents. Activities that belong to QSEs pertaining to restoring service such as customer management and continual improvement were ascribed to Phase 3 (Fig. 2).

For implementation, we developed tools and integrated them with response and recovery activities^1^. These tools have been made freely available under a Creative Commons Attribution (CC-BY 4.0) license online to assist with enhancing laboratory preparedness efforts (https://figshare.com/s/4f9bf87e55304b5ae577). An important aspect of a quality systems approach to disaster recovery is the identification and implementation of Quality Indicator (QI) assessment^2^. QIs provide an objective measure to potentially evaluate all critical domains identified in the disaster recovery process. Data from and outcomes associated with those domain assessments may be implemented in a consistent and comparable manner across settings and over time. When assessing the quality of restored laboratory services using QIs, it is important to ensure systematic and consistent data collection and analysis by using a comprehensive set of indicators that addresses all stages of the assessment^2^.

## Phase 1: Laboratory assessments

The impact on Hurricane Maria on the PRDH PHL’s ranged from tangible, e.g. roof damage, to intangible, including disruption of internal laboratory processes and procedures that are critical for providing services. In Phase 1, after assuring the safety of all the personnel, facilities were assessed to systematically identify the areas of impact and the organization of the response was established. Laboratory assessments were conducted by an interdisciplinary team of CDC laboratory scientists with experience in management of laboratory quality, safety, and CLIA-regulated testing, who represented a variety of subject matter expertise areas. Team members were recruited for overlapping 3-week deployments, in an approach that facilitated a flexible, rapidly mobilizable and sustainable emergency response. The first representatives of the team deployed to Puerto Rico on 12 October 2017, in recognition that rapid needs assessment is foundational to strategic response. Deployment timing was impacted by logistical constraints due to limited access to the island following a natural disaster of the scale of Hurricane Maria. Assessment of laboratory spaces was triaged based on the identification of priority pathogens, as determined by PRDH SME’s. Assessments of spaces critical for restoring testing capacity for these pathogens were completed within 2 weeks of the first deployment. Deployers used an assessment tool previously developed for the baseline assessment of foodborne, waterborne, and environmental laboratory activities. The tool also contained questions about accreditation, licensing, quality and additional questions covering CLIA activities were also added. Subsequent members of the laboratory team assessed remaining laboratory spaces, including those at other PRDH facilities across the island, throughout the month of November. In the weeks following Hurricane Maria, CDC asked the Association of Public Health Laboratories (APHL) to conduct independent assessments of the PRDH facilities during the month of October. Assessments of PRDH facilities were also conducted by the Federal Emergency Management Agency (FEMA).

**Fig. 3:**
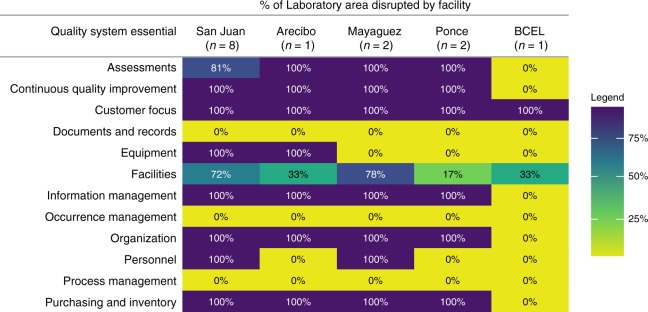
A heatmap showing the results of the initial assessment by QSE. Note the assessment was conducted for the distinct laboratory areas, and some units work in a shared laboratory space. Each QSE encompasses multiple subdomains; percentage disruption for each laboratory was estimated by calculating the percentage of these subdomains that were impacted by Hurricane Maria

CDC findings were classified based on the QSE framework, which is a conceptual scaffolding that represents the elements of a laboratory quality management system (Table 1 and Fig. 3)^3^. While the individual findings from APHL and CDC assessment teams were consistent, this quality-focused approach was developed by CDC deployers to address the need for a prospective, systems-wide evaluation of the impact of the hurricane to facilitate recovery of this complex laboratory network. Multiple barriers to re-establishment of services were identified in the aftermath of Hurricane Maria, including a need for access to uninterrupted municipal power, facility damage, damage to critical equipment and reagents, and challenges with procurement. The Biological and Chemical Emergency Laboratory (BCEL) and Newborn Screening Laboratory sustained minimal damage and service interruptions (Jessica Cabrera, PRDH personal communication). However, two laboratory facilities (in San Juan and Mayaguez, see Fig. 1) sustained substantial structural damage. Across the four affected PRDH public health laboratory facilities, nine (64%) of the 14 individual laboratories reported testing disruption.

**Table 1:** Laboratory needs identified by initial assessments

QSE		San Juan (*n* = 8)	Arecibo (*n* = 1)	Mayaguez (*n* = 2)	Ponce (*n* = 2)	Overall %
Facilities						
	Damaged, accessible facilities	2	0	1	1	31
	Damaged, inaccessible facilities	4	0	1	0	38
	Service disruption: electricity	8	1	2	0	85
	Service disruption: air conditioning	8	0	2	0	77
	Service disruption: bathrooms	0	0	0	0	0
	Service disruption: telecommunications	8	1	2	2	100
	Service disruption: air quality	6	1	2	0	69
	Service disruption: waste management	8	0	2	0	77
	Service disruption: security	8	0	2	0	77
Organization						
	Disruption to management and administration	8	1	2	2	100
Personnel						
	labs with at least one staff member facing certification loss	5	1	2	2	77
Equipment						
	Damaged essential equipment	8	1	0	0	69
Purchasing and inventory						
	Service disruption: procurement	8	1	2	2	100
	Damaged reagents	8	1	2	1	92
Process management						
	Checklists and procedures damaged	0	0	0	0	0
Information management						
	Service disruption: data transfers	8	1	2	0	85
Documents and records						
	Service disruption: information management	0	0	0	0	0
Customer focus						
	Service disruption: diagnostic and surveillance testing	8	1	2	0	85
Assessments						
	Service disruption: external assessments (e.g., proficiency programs)	5	1	2	2	77
	Service disruption: internal assessments (e.g., quality control programs)	8	1	2	2	100
Occurrence management						
	Service disruption: nonconforming events management	0	0	0	0	0
Continuous quality improvement						
	Service disruption: quality indicators (based on proficiency testing)	8	1	2	2	100

Laboratories within facility (number)

Consolidated overview of laboratory needs identified after initial assessments following Hurricane Maria. The laboratory needs are categorized by quality system essential (QSE)

This QSE-based approach facilitated the integration of prospective laboratory assessments with response activities, through the identification and monitoring of inter-dependent laboratory needs. Initial assessments of the laboratory spaces showed that direct damage from water or wind, and indirect damage due to power fluctuations following the storm and subsequent attempts to connect the buildings to generators (Table 1 and Fig. 3), impacted laboratory equipment. In addition to analytical instruments used for diagnostic, surveillance and environmental testing, disruptions were documented for safety equipment (e.g., chemical fume hoods and biosafety cabinets) and support equipment (e.g., autoclaves, incubators, refrigerators).

As a result of impacted cold storage 12 (86%) of the individual laboratories reported loss of critical reagents. Reagents stored at room temperature also have temperature range requirements, which were challenging to maintain without climate control. Therefore, an initial priority was to mitigate critical laboratory needs in the facilities and safety domain, followed by procurement of reagents and tools necessary for qualification of equipment (Fig. 2).

A critical intangible impact of Hurricane Maria on PHL capacity was the status of laboratory certifications. PRDH laboratories are certified by several agencies. Human testing is regulated by CMS, milk testing by the Food and Drug Administration (FDA) and water testing by the Environmental Protection Agency (EPA). Proficiency testing, a method of evaluation by an external regulatory body based on the ability of a laboratory to evaluate a panel of standardized unknown specimens, is a cornerstone of laboratory certification. Laboratory Team members worked with PRDH laboratory SMEs to understand the impact of Hurricane Maria on proficiency and certification timelines, which could potentially result in regulatory non-compliance. This phased approach informed assessment of reagent needs to support quality assurance of clinical, surveillance and environmental testing (e.g., proficiency panels, calibration standards, and experimental controls). In turn, the reactivation of proficiency testing services administered by PRDH had important implications for other public health and clinical laboratories within the jurisdiction, which rely on PRDH central PHL’s to support their own quality assurance programs.

## Phase 2: Rapid procurement

In Phase 2, by controlling the process of how equipment was excessed (if damaged) or purchased and inventoried, the response moved quickly into the next phase of restoring local diagnostic and surveillance testing. Maintaining documents and records was key to ensure items were received and qualified and that service and maintenance contracts were procured. Hurricane Maria also impacted procurement. The laboratory team worked with PRDH SME’s to identify vendors (including local vendors); create purchasing accounts when necessary; verify prices; set the terms of contracts and service agreements; and finally, purchase the goods. The only two delivery routes to Puerto Rico are by air and by sea. Because of disruption in routine shipping systems, optimization of these procedures (e.g., minimizing freight charges by grouping equipment orders) was a strategic element of the process.

**Fig. 4:**
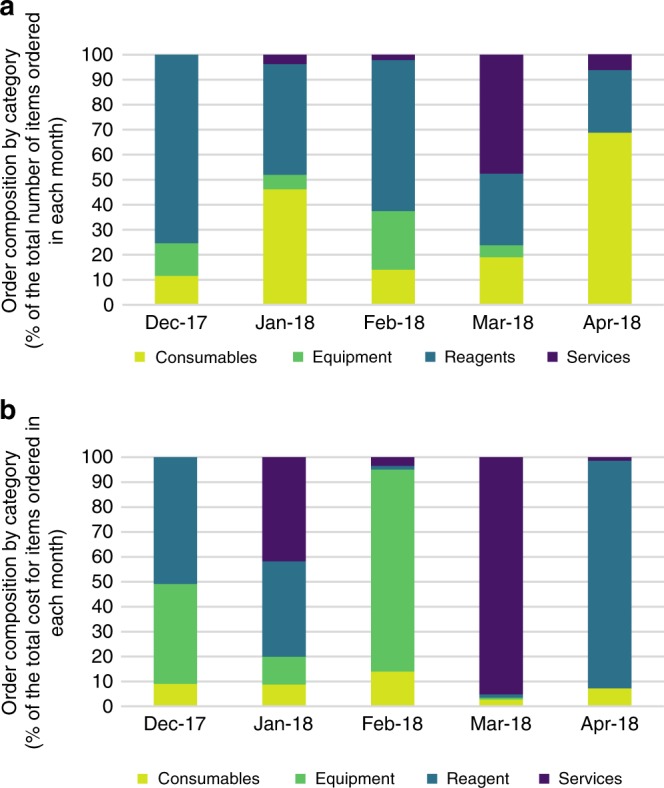
Procurement for PRDH laboratory needs (includes all facilities). Procurement was classified by product type and % of the total number (**a**) or cost (**b**) of items ordered. Items were grouped into the following categories: consumables (e.g., plasticware, shipping and packing materials, office supplies); equipment (e.g., refrigerators, freezers, pH meters); reagents (e.g., buffers, reagents, calibration standards); services (e.g., repair, installation, certification, calibration, preventative maintenance contracts; in many cases, more than one instrument was serviced at a time)

To expedite procurement of laboratory supplies and bring diagnostic and environmental testing services and surveillance activities back online in Puerto Rico, the CDC Laboratory Team partnered with the CDC Foundation. The CDC Foundation is an independent, nonprofit organization, authorized by Congress to mobilize philanthropic partners and private-sector resources to support CDC’s critical health protection mission^4^. The first shipments included essential laboratory items (consumables, reagents, and equipment needed to reestablish testing). Later orders included laboratory items that failed quality control checks when PRDH was returned to grid power, as well as those that failed after prolonged exposure to heat and humidity (Fig. 4). Accordingly, the majority of services needed to calibrate or validate equipment were procured later in the response (Fig. 4). By June 2018, 72% of the orders had been delivered; 16% had been ordered but not yet delivered; and 12% were pending (because items were backordered, discontinued, or otherwise no longer available). The identification and procurement of essential supplies, including reagents, proficiency materials, and equipment were collaboratively achieved by laboratory SMEs at PRDH and the CDC Laboratory Team.

**Fig. 5:**
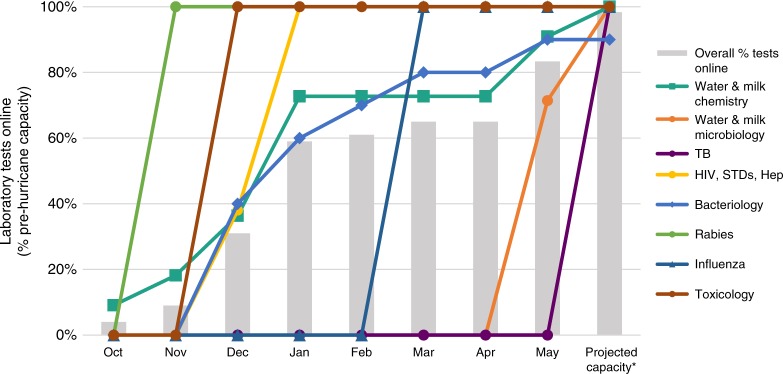
PRDH laboratory testing capacity as of 18 May 2018. Capacity is presented as a percent of the actual volume of tests typically conducted by each laboratory in the previous year (2016). By this date 92% of the tests by volume were operational; by the number of tests offered before Hurricane Maria, 98% of the total testing capacity was reestablished. The “Projected Capacity*” column indicates the adjusted laboratory testing status after pending repairs were completed

As of 31 March 2018, 178 days into the laboratory response, the influenza, HIV, and rabies testing laboratories were fully operational; other laboratories, including bacteriology, were functioning at ~59% of their testing capacity (based on previous year volume of testing). By 18 May 2018, 226 days into the laboratory response, 92% of the laboratory testing capacity based on the volume of tests run in the previous year could be performed (Fig. 5). An inventory template for documenting relevant information for essential laboratory supplies is provided at https://figshare.com/s/4f9bf87e55304b5ae577.

## Phase 3: Enhanced quality management to reestablish laboratory testing

Phase 3 was particularly important because the sequential handover to local partners occurred during this time; the timeline of the transition from phase 2 to phase 3 varied by facility and by subject matter area. In this phase, managing the information and communicating with stakeholders (including internal and external partners) was key. If nonconforming events occur, they should be managed and used to inform the continual improvement process.

In addition to procurement of critical laboratory supplies, infectious disease testing needed to be reestablished. During the initial response, priority pathogens were identified by PRDH and partner laboratories in the continental US were identified by the CDC Laboratory Team to reestablish interim clinical diagnostic testing and surveillance for those pathogens starting in October 2017. Sample shipping and tracking systems were implemented by the CDC Laboratory Team to facilitate this process and provide quality assurance^5^. A template for a sample shipping and tracking database is provided at https://figshare.com/s/4f9bf87e55304b5ae577. In December 2017, two QSE modules covering sample shipping and sample tracking were developed. Analysis of QI, e.g. average sample shipping time (defined as the time a package left PRDH in San Juan until it was received by CDC in Atlanta) from 1.3 days in November to 1.0 days in December demonstrated the impact of tool implementation. The shipping time remained at less than 1.1 days through the duration of the response (until May 2018), with one exception due to adverse weather events in January 2018 that affected air traffic nationwide. The primary reason for improved shipping was due to the reduction in the number of packages delayed or rejected by the courier because of errors in package labeling. As of 18 May 2018, more than 2800 clinical and surveillance samples were shipped from PRDH to partner testing laboratories. CDC tested 75% of the samples shipped, and coordinated the shipping of samples being tested for tuberculosis to partner laboratories in the states of Georgia, Virginia, and Florida. The CDC Foundation and partner organizations like APHL and CDC EOC supplied necessary items to facilitate the sample shipping process, including courier fees and laboratory consumables like biohazard bags, shipping boxes, dry ice, and ice packs.

The PRDH facilities that provided testing for priority pathogens were the initial focus, although the overlap in laboratory facilities meant that many of the laboratory concerns were addressed simultaneously. Additional requirements to reestablish laboratory operations included reconnection to the electrical power grid, repair or replacement of air conditioning units, repair of back-up electric generators, mold remediation, re-certification/re-qualification of essential equipment, and further support for existing documentation. Hurricane Maria resulted in extensive losses of high-cost, high-impact electronic laboratory equipment, including analytical instrument computers, printers, cables, and local network/Internet access. One crucial recovery measure implemented was the installation of uninterruptible power source (UPS) towers, which allowed for a controlled shutdown of instruments when generator or grid power was interrupted. Achieving full power status (defined as a stable connection to grid power and functional air conditioning) in the laboratory areas (excluding administrative and office spaces) was coordinated between federal agencies and PRDH. By mid-February 2018, the percent of laboratory areas that achieved full power status increased to more than 80%.

A final consideration for the recovery phase was the expansion of the QMS already in place at PRDH. The existing QMS provided support toward long-term recovery and complemented PRDH’s emergency preparedness and readiness plans. With the influx of new refrigerators, freezers, and incubators, attendant National Institute of Standards and Technology (NIST)-certified thermometers were needed. Ordering new equipment required measuring available space and making sure surfaces were leveled. Basic items such as a tape measure and a level to perform these tasks were not easily available. It was quickly realized that an Emergency Laboratory Kit needed to be developed that included components required to move or re-qualify equipment and establish whether instruments needed to be excessed and replaced. The collection of equipment listed in the Example Emergency Laboratory Kit that can be found at https://figshare.com/s/4f9bf87e55304b5ae577 helped us establish priorities for equipment repair or replacement.

## Conclusions

PHLs are a critical component of emergency public health responses in general. Laboratory activities are integral to confirming environmental, epidemiological and clinical investigations for individuals and vulnerable populations following an emergency event. In order to appropriately respond, health professionals and responders need diagnoses for individual patients as well as syndromic surveillance to detect outbreaks or emerging diseases.

The mitigation tools developed and available at https://figshare.com/s/4f9bf87e55304b5ae577 were used during the response described here to help reestablish laboratory testing capacity to 92% within a period of 6 months. Some of the local routine testing at PRDH was resumed starting in December 2017, when the laboratories testing for rabies and influenza surveillance were restored and recertified. The sequential handover was accomplished collaboratively by PRDH staff and the CDC Laboratory Team as laboratory spaces were restored, with assistance from the multidisciplinary partners (including CDC field staff assigned to PRDH) who worked closely across federal agency jurisdictions and geographic boundaries. The reestablishment of testing illustrated in Fig. 5 highlights the timing of reintegration into a routine system. After Hurricane Maria, the impact of the interruption of laboratory services on CLIA-compliant testing was mitigated in consultation with the CMS agency, which issued a temporary waiver for proficiency testing; in February 2018, PRDH passed its semiannual CMS survey for CLIA recertification with no deficiencies.

An additional outcome of this work has been the enhancement of the preparedness of public health laboratory facilities by creating tools to facilitate the reestablishment of services after interruption by an emergency event. Logistical challenges addressed included equipment and reagent delivery delays due to interruption in the transportation chain, and the installation of larger items when building infrastructure was damaged (e.g., freight elevators). The resources and templates created during this response are in the public domain, and are available for download at https://figshare.com/s/4f9bf87e55304b5ae577. The Inventory and Priority Supplies Template and the Sample Shipping and Tracking Log Template are available for laboratory staff and administration to review, edit as appropriate, and implement to help secure their own facilities to support enterprise level preparedness activities. We have also developed a training manual for laboratory responders which proved useful to maintain continuity while deployed staff rotate through, and are demobilized and backfilled. By using a quality management-based, process control approach, the workflows, and templates presented in this Perspective are widely applicable to public health and other laboratory networks that could be damaged by a regional disaster event, or in other emergency circumstances when PHL facilities are compromised or stressed.

## Data Availability

All data is available in the manuscript or in FigShare (https://figshare.com/s/4f9bf87e55304b5ae577).
